# Genome Sequence of *Brevibacillus formosus* F12^T^ for a Genome-Sequencing Project for Genomic Taxonomy and Phylogenomics of *Bacillus*-Like Bacteria

**DOI:** 10.1128/genomeA.00753-15

**Published:** 2015-07-23

**Authors:** Jie-Ping Wang, Bo Liu, Guo-Hong Liu, Qian-qian Chen, Yu-jing Zhu, Zheng Chen, Jian-mei Che

**Affiliations:** Agricultural Bio-Resources Research Institute, Fujian Academy of Agricultural Sciences, Fuzhou, Fujian, China

## Abstract

*Brevibacillus formosus* F12^T^ is a Gram-positive, spore-forming, and strictly aerobic bacterium. Here, we report the draft 6.215-Mb genome sequence of *B. formosus* F12^T^, which will provide useful information for genomic taxonomy and phylogenomics of *Bacillus*-like bacteria, as well as for the functional gene mining and application of *B. formosus*.

## GENOME ANNOUNCEMENT

The genus *Brevibacillus* within the family *Paenibacillaceae* was established by Shida et al. in 1996 (1). Up to now, a total of 20 validly nominated species have been assigned taxonomically to this genus (http://www.bacterio.net/brevibacillus.html). It is worth mentioning that nine species (namely, *B. brevis*, *B. agri*, *B. borstelensis*, *B. centrosporus*, *B. choshinensis*, *B. formosus*, *B. laterosporus*, *B. reuszeri*, and *B. thermoruber*) were reclassified from the genus *Bacillus* on the basis of a 16S rRNA gene sequence analysis (1). More interestingly, except *Bacillus brevis* (2) and *Bacillus thermoruber* (3), the other seven reclassified *Bacillus* species were all identified from different strains of *B. brevis* by three independent taxonomic studies of *B. brevis* strains (4–6).

Because of the above-mentioned taxonomic history and no available genomic information for *B. formosus*, its type strain, F12^T^, was selected as one of the research objects in our genome-sequencing project for genomic taxonomy and phylogenomics of *Bacillus*-like bacteria. The strain F12^T^ was deposited in seven culture collection organizations under the following numbers: ATCC 51669, CIP 104544, DSM 9885, IFO (now NBRC) 15716, JCM 9169, LMG 16010, and NRRL NRS-863. Furthermore, it was demonstrated that some strains of *B. formosus* exhibited promising application prospects. *B. formosus* BISR-1, isolated from the Great Indian Desert soils, has biocontrol potential against phytopathogenic fungi and can produce a hyperthermostable chitinase that retains a half-life of >5 h at 100°C (7). *B. formosus* BN53-1 was found to possess the potential to treat livestock and poultry feces for odor control due to its hydrogen sulfide-decomposing activity (8).

The genome sequencing of *B. formosus* F12^T^ was performed via the Illumina HiSeq 2500 system. Two different DNA libraries with insert sizes of 500 and 3,000 bp were constructed and sequenced. After filtering of the 1.23 Gb of raw data, the 1,008-Mb clean sequence data were obtained, providing approximately 150-fold coverage. The reads were assembled via the SOAP*denovo* software version 1.05 (9). Through the data assembly, 26 scaffolds consisting of 6,215,362 bp were obtained, and the scaffold *N*_50_ was 759,282 bp. The average length of the scaffolds was 239,052 bp, and the longest and shortest scaffolds were 1,166,708 bp and 1,270 bp, respectively. Moreover, 93.00% clean reads were aligned back to the genome, by which 99.95% of the genome sequence was covered.

Annotation of the genome was performed using the NCBI Prokaryotic Genomes Automatic Annotation Pipeline (PGAAP) utilizing the GeneMark, Glimmer, and tRNAscan-SE tools (10). A total of 5,765 genes were predicted, including 5,486 coding sequences (CDS), 118 pseudogenes, 152 tRNAs, and 8 rRNA genes. There were 4,013 and 2,497 genes assigned to COG and the KEGG database, respectively. Also, one clustered regularly interspaced short palindromic repeat (CRISPR) array was found in the draft genome. The average DNA G+C content was 47.42%, being compatible with the value 47.2 mol% acquired by high-performance liquid chromatography (HPLC) determination (5).

### Nucleotide sequence accession numbers.

This whole-genome shotgun project has been deposited at DDBJ/EMBL/GenBank under the accession no. LDCN00000000. The version described in this paper is version LDCN01000000.
